# Genetic diversity and virulence properties of caprine *Trueperella pyogenes* isolates

**DOI:** 10.1186/s12917-024-04262-x

**Published:** 2024-09-06

**Authors:** Ewelina Kwiecień, Ilona Stefańska, Magdalena Kizerwetter-Świda, Dorota Chrobak-Chmiel, Michał Czopowicz, Agata Moroz-Fik, Marcin Mickiewicz, Kinga Biernacka, Emilia Bagnicka, Jarosław Kaba, Magdalena Rzewuska

**Affiliations:** 1 Department of Preclinical Sciences, Institute of Veterinary Medicine, Warsaw University of Life Sciences, Ciszewskiego 8 St, 02-786 Warsaw, Poland; 2 Division of Veterinary Epidemiology and Economics, Institute of Veterinary Medicine, Warsaw University of Life Sciences, Nowoursynowska 159C St, 02-776 Warsaw, Poland; 3 Institute of Genetics and Animal Biotechnology, Polish Academy of Sciences, Postępu 36A St, 02-552 Jastrzębiec, Magdalenka Poland

**Keywords:** Biofilm, Genetic diversity, Goats, RAPD-PCR, Trueperella pyogenes, Virulence genotypes

## Abstract

**Background:**

*Trueperella pyogenes* is an opportunistic pathogen that causes suppurative infections in various animal species, including goats. So far, only limited knowledge of phenotypic and genotypic properties of *T. pyogenes* isolates from goats has been gathered. In our study, we characterized the phenotypic and genotypic properties of caprine *T. pyogenes* isolates and established their relationship by Random Amplified Polymorphic DNA-Polymerase Chain Reaction (RAPD-PCR).

**Results:**

From 2015 to 2023, 104 T*. pyogenes* isolates were obtained from 1146 clinical materials. In addition, two *T. pyogenes* isolates were obtained from 306 swabs collected from healthy goats. A total of 51 T*. pyogenes* isolates were subjected to detailed phenotypic and genotypic characterization. The virulence genotype *plo/nanH/nanP/fimA/fimC/luxS* was predominant. All of the tested isolates showed the ability to form a biofilm but with different intensities, whereby most of them were classified as strong biofilm formers (72.5%). The high level of genetic diversity among tested caprine *T. pyogenes* isolates (19 different RAPD profiles) was observed. The same RAPD profiles were found for isolates obtained from one individual, as well as from other animals in the same herd, but also in various herds.

**Conclusions:**

This study provided important data on the occurrence of *T. pyogenes* infections in goats. The assessment of virulence properties and genetic relationships of caprine *T. pyogenes* isolates contributed to the knowledge of the epidemiology of infections caused by this pathogen in small ruminants. Nevertheless, further investigations are warranted to clarify the routes of transmission and dissemination of the pathogen.

**Supplementary Information:**

The online version contains supplementary material available at 10.1186/s12917-024-04262-x.

## Background

*Trueperella pyogenes* is a Gram-positive bacterium and a common inhabitant of the skin and the mucous membranes of the upper respiratory, gastrointestinal, and urogenital tract of animals. *T. pyogenes* may also be pathogenic for livestock and wild animals, causing different purulent infections, including mastitis, metritis, pneumonia, and abscesses of various locations [1]. Studies on infections caused by this opportunistic pathogen mainly focused on cattle and pigs. The information about *T. pyogenes*-induced infections in other animal species is fairly limited. Nevertheless, the pathogenic potential of *T. pyogenes* in relation to other hosts should not be overlooked. *T. pyogenes* infections in pigs and cattle generate significant economic losses [2], but little is known about the occurrence of diseases caused by this pathogen in small ruminants. According to the available data, in goats, *T. pyogenes* has been isolated from abscesses of various organs and tissues [2–6] as well as from udder lesions [2].

Several virulence factors related to the pathogenic potential of *T. pyogenes* are known. Cytotoxin, pyolysin (PLO), is considered the main one. Moreover, factors associated with adhesion and colonization of host tissues, such as neuraminidase H and P (NanH and NanP), several types of fimbriae, including FimA, FimC, FimE and FimG, as well as collagen-binding protein (CbpA), are also involved in the pathogenicity of this bacterium [1]. The knowledge of *T. pyogenes* virulence comes mainly from studies on bovine and swine isolates [3, 4, 7, 8], and data regarding isolates of other origins are limited. The evaluation of virulence profiles is a very important aspect of the characterization of bacterial isolates. Moreover, the pathogenicity of this bacterium may also depend on the capability to form biofilm [8, 9]. This property is particularly significant because it may be a hindrance in the antimicrobial therapy of *T. pyogenes* infections.

In the case of pathogenic bacteria, apart from phenotypic and genotypic characterization, another important aspect is the determination of genetic diversity and relationship among isolates, which may be associated with the pathogenic potential of the bacteria. So far, some molecular typing methods have been used to study the genetic diversity of *T. pyogenes* strains isolated from different origins [9–14], but only little data on the genetic differentiation of caprine *T. pyogenes* isolates are available [13, 14].

The aim of this study was to assess the presence of virulence factors and biofilm formation capability, as well as the genetic diversity of caprine *T. pyogenes* isolates. Additionally, the carriage of *T. pyogenes* in clinically healthy goats was investigated.

## Results

### *T. pyogenes* isolates from infections in goats

*T. pyogenes* was isolated from 104/1146 (9.1%; CI 95%: 7.5%-10.9%) studied clinical specimens collected from diseased goats. The isolates were obtained from: udder (*n* = 12), vagina (*n* = 7), lymph nodes (*n* = 12), lungs (*n* = 31), bronchus (*n* = 16), trachea (*n* = 1), ear (*n* = 1), and abscess of different locations (*n* = 24) (Table 1). In 91/104 (87.5%; CI 95%: 79.8%-92.5%) cases, *T. pyogenes* was isolated as a single pathogen. In the remaining 13/104 (12.5%; CI 95%: 7.5%-20.2%) cases, *T. pyogenes* co-infections with *Mannheimia haemolytica* (*n* = 4), *Staphylococcus aureus* (*n* = 3), *Escherichia coli* (*n* = 2), *Pasteurella multocida* (*n* = 2), *Corynebacterium pseudotuberculosis* (*n* = 1), and *Rhodococcus equi* (*n* = 1) were observed. Of 104 T*. pyogenes* isolates obtained from infected animals, 49 were retained for future analysis, whereas 55 were disposed of.

**Table 1:** Origin and virulence genotypes of caprine *T. pyogenes* isolates tested in this study (*n* = 51)

Isolate designation^*^	Isolation site	Year of isolation	Virulence genotype	RAPD profile
12/K	udder	2016	*plo/nanH/nanP/fimA/fimC/luxS*	R10.3
13/K^1^	lung	2016	*plo/nanH/nanP/fimA/fimC/luxS*	R10.3
14/K^1^	vagina	2016	*plo/nanH/nanP/fimA/fimC/luxS*	R10.3
15/K	vagina	2016	*plo/nanH/nanP/fimA/fimC/luxS*	R10.3
16/K^1^	bronchus	2016	*plo/nanH/nanP/fimA/fimC/fimE/luxS*	R10.3
17/K	vagina	2016	*plo/nanH/nanP/fimA/fimC/fimE/luxS*	R10.3
18/K^2^	abdomen	2016	*plo/nanH/nanP/fimA/fimC/fimE/luxS*	R7.2
23/K^3^	bronchus	2015	*plo/nanH/nanP/fimA/fimE/luxS*	R10.2
24/K	lung	2015	*plo/nanH/nanP/fimC/luxS*	R10.2
25/K^3^	lung	2015	*plo/nanH/nanP/fimC/luxS*	R10.3
29/K	lung	2016	*plo/nanH/nanP/fimC/fimE/luxS*	R8.1
32/K^4^	vagina	2016	*plo/nanH/nanP/fimC/fimE/luxS*	R9.2
33/K	lung	2016	*plo/nanH/nanP/fimA/fimC/luxS*	R10.3
35/K^4^	lung	2016	*plo/nanH/nanP/fimC/fimE/luxS*	R5
50/K	abscess	2016	*plo/nanH/nanP/fimA/fimC/fimE/luxS*	R3
55/K^2^	udder	2016	*plo/nanH/nanP/fimA/fimE/luxS*	R7.2
57/K	abscess	2015	*plo/nanH/nanP/fimA/fimE/luxS*	R1
58/K	abscess	2015	*plo/nanH/nanP/fimA/fimC/luxS*	R9.2
59/K	lung	2016	*plo/nanH/nanP/fimA/fimC/fimE/luxS*	R10.1
60/K	lung	2016	*plo/nanH/nanP/fimA/fimC/fimE/luxS*	R10.2
61/K	lung	2016	*plo/nanH/nanP/fimA/fimC/fimE/luxS*	R6.2
62/K	skin	2017	*plo/nanH/nanP/fimA/fimC/luxS*	R9.2
63/K	mandible	2017	*plo/nanH/nanP/fimA/fimC/fimE/luxS*	R1
64/K	bronchus	2017	*plo/nanH/nanP/fimA/fimC/luxS*	R9.2
65/K	bronchus	2017	*plo/nanH/nanP/fimA/fimC/fimE/luxS*	R10.3
66/K	lung	2018	*plo/nanH/nanP/fimA/fimC/luxS*	R8.3
67/K	lung	2018	*plo/nanH/nanP/fimA/fimC/luxS*	R9.2
68/K	lung	2018	*plo/nanH/nanP/fimA/fimE/luxS*	R7.1
69/K	lung	2018	*plo/nanH/nanP/fimA/fimC/luxS*	R8.2
72/K^5^	lung	2018	*plo/nanH/nanP/fimA/fimC/luxS*	R2
73/K^5^	lung	2018	*plo/nanH/nanP/fimA/fimC/luxS*	R2
74/K^5^	bronchus	2018	*plo/nanH/nanP/fimA/fimC/luxS*	R2
75/K^5^	bronchus	2018	*plo/nanH/nanP/fimA/fimC/luxS*	R2
76/K^6^	lung	2019	*plo/nanH/nanP/fimA/fimC/fimE/luxS*	R9.1
77/K^6^	udder	2019	*plo/nanH/nanP/fimA/fimC/fimE/luxS*	R9.1
78/K^6^	udder	2019	*plo/nanH/nanP/fimA/fimC/fimE/luxS*	R9.1
79/K	abscess	2019	*plo/nanH/nanP/fimA/fimC/fimE/luxS*	R10.2
80/K	nasal cavity	2018	*plo/nanH/nanP/fimA/fimC/luxS*	R10.2
81/K	nasal cavity	2018	*plo/nanH/nanP/fimC/luxS*	R10.1
82/K	udder	2020	*plo/nanH/nanP/fimA/fimC/luxS*	R9.2
83/K	lung	2020	*plo/nanH/nanP/fimC/luxS*	R10.2
84/K	mediastinal lymph node	2020	*plo/nanH/nanP/fimC/luxS*	R10.2
85/K	abscess	2021	*plo/nanH/nanP/fimA/fimC/fimE/luxS*	R1
86/K	udder	2021	*plo/nanH/nanP/fimA/fimC/fimE/luxS*	R4.1
87/K	abscess	2022	*plo/nanH/nanP/fimA/fimC/fimE/luxS*	R6.2
88/K	abscess	2022	*plo/nanH/nanP/fimA/fimC/luxS*	R6.1
89/K^7^	lung	2022	*plo/nanH/nanP/fimA/fimC/luxS*	R9.3
90/K^7^	mediastinal lymph node	2022	*plo/nanH/nanP/fimA/fimC/luxS*	R9.3
91/K^7^	trachea	2022	*plo/nanH/nanP/fimA/fimC/luxS*	R9.3
92/K	abscess	2022	*plo/nanH/nanP/fimA/fimC/fimE/luxS*	R4.2
93/K	abscess	2022	*plo/nanH/nanP/fimA/fimC/fimE/luxS*	R4.2

^1^^−^^7^* T. pyogenes* isolates from the same goats but from different clinical samples

### *T. pyogenes* isolates from clinically healthy animals

*T. pyogenes* was isolated from 2/306 (0.7%; CI 95%: 0.2%-2.4%) swabs collected from clinically healthy goats and this proportion was significantly lower than in diseased goats (*p* < 0.001). Both isolates were obtained from nasal swabs taken from two goats from the same herd (I) (isolate 80/K and 81/K).

### Phenotype of *T. pyogenes* isolates

Finally, the total of 51 T*. pyogenes* isolates from goats were included in the study. Among them, 49 isolates were obtained from purulent lesions or abscesses in various tissues, and two isolates were isolated from the nasal cavity of clinically healthy goats. The accurate origin description of studied isolates is presented in Table 1, including site and year of isolation. Tested isolates were isolated from 39 goats – 21 kept in herd I, two in herd II, and three in herd III. The animal origin (herd and/or goat number) was unknown for some isolates.

All *T. pyogenes* isolates showed cell morphology typical of this species (small, white colonies with the β-hemolysis zone), and were Gram-positive irregular rods, characterized by negative catalase test. In the Christie–Atkins–Munch-Peterson (CAMP) test, all isolates showed a synergistic effect of the enhanced hemolysis with the *S. aureus* ATCC®25,923 reference strain, but with varying intensity [data not shown].

### Biofilm production in vitro

All tested caprine *T. pyogenes* isolates (*n* = 51) showed the ability to form a biofilm but with different intensities (Table 2). Most of them (*n* = 37; 72.5%, CI 95%: 59.1%-82.9%) were strong biofilm formers. Moderate and weak biofilm formers comprised 19.6% (CI 95%: 11.0%-32.5%; *n* = 10) and 7.8% (CI 95%: 3.1%-18.5%; *n* = 4), respectively. The absorbance values obtained for particular caprine *T. pyogenes* isolates are shown in Additional file 1.

**Table 2:** Biofilm formation by caprine *T. pyogenes* isolates (*n* = 51) according to the crystal violet staining method

Mean OD value^*^	Number of isolates (%)	Biofilm formation
OD_570_ ≤ 0.091	0 (0)	No
0.091 < OD_570_ ≤ 0.182	4 (7.8)	Weak
0.182 < OD_570_ ≤ 0.364	10 (19.6)	Moderate
0.364 < OD_570_	37 (72.6)	Strong

^*^Optical density cut-off value (OD_c_) = mean OD of the negative control + 3 × standard deviation (SD) of the negative control; (negative control – sterile TSB); OD_c_ = 0.091

### Virulence genes

All *T. pyogenes* isolates (*n* = 51) carried the *plo*, *nanH*, *nanP* and *luxS* genes. The *fimA*, *fimC* and *fimE* genes were detected in 43/51 (84.3%; CI 95%: 72.0%-91.8%), 47/51 (92.2%; CI 95%: 81.5%-96.9%), and 25/51 (49.0%; CI 95%: 35.9%-62.3%) isolates, respectively. Among tested isolates, 18/51 (35.3%; CI 95%: 23.6%-49.0%), 28/51 (54.9%; CI 95%: 41.4%-67.7%), and 5/51 (9.8%; CI 95%: 4.3%-21.0%) carried simultaneously three, two and single genes encoding fimbriae subunits, respectively. None of the tested *T. pyogenes* isolates carried the *cpbA* and *fimG* genes (Table 1).

Among tested caprine *T. pyogenes* isolates, five different virulence genotypes were identified. The dominant genotype, detected in 21/51 (41.2%; CI 95%: 28.8%-54.8%) isolates, was *plo/nanH/nanP/fimA/fimC/luxS*. In the remaining 30/51 (58.8%; CI 95%: 45.2%-71.2%) isolates, virulence genes were present in the following combinations: *plo/nanH/nanP/fimA/fimC/fimE/luxS* (18/51; 35.3%; CI 95%: 23.6%-49.0%), *plo/nanH/nanP/fimA/fimE/luxS* (4/51; 7.8%; CI 95%: 3.1%-18.5%), *plo/nanH/nanP/fimC/fimE/luxS* (3/51; 5.9%; CI 95%: 2.0%-15.9%), *plo/nanH/nanP/fimC/luxS* (5/51; 9.8%; CI 95%: 4.3%-21.0%). The data about virulence genotypes among the tested caprine *T. pyogenes* isolates are presented in Table 1.

### RAPD profiles and relationships of caprine *T. pyogenes* isolates

**Fig. 1:**
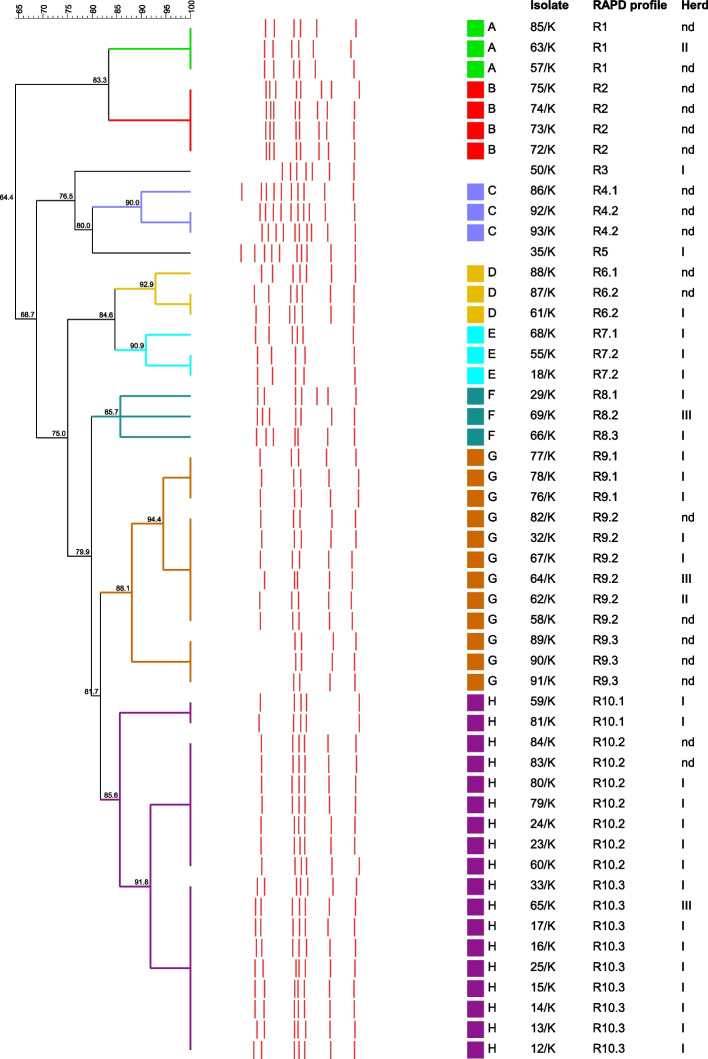
Dendrogram designed based on the results of RAPD-PCR typing of 51 caprine *T. pyogenes* isolates using UPGMA analysis and Dice correlation coefficient. Eight clusters designated from A to H were based on the similarity cut-off of 85%. *T. pyogenes* isolates from the same goats but from different clinical specimens are 13/K, 14/K, and 16/K; 18/K and 55/K; 23/K and 25/K; 32/K and 35/K; 72/K, 73/K, 74/K, and 75/K; 76/K, 77/K, and 78/K; 89/K, 90/K, and 91/K. In the column ʺherdʺ, the ʺndʺ means not data

Nineteen different RAPD profiles were distinguished among the 51 tested caprine *T. pyogenes* isolates. The analysis showed the most prevalent RAPD profiles, R10.3, R10.2 and R9.2, grouped nine, seven and six isolates, respectively. The RAPD profile R2 grouped four isolates. Three RAPD profiles, R1, R9.1 and R9.3, grouped three isolates each, and RAPD profiles, R4.2, R6.2, R7.2 and R10.1, grouped two isolates each. Eight *T. pyogenes* isolates presented unique RAPD profiles. Some tested isolates were obtained from the same individuals but from various clinical specimens. In these cases, the majority of isolates from the same goats belonged to the same RAPD profiles, as follows: 72/K, 73/K, 74/K, and 75/K to the R2 RAPD profile, 18/K and 55/K to the R7.2 RAPD profile, 76/K, 77/K, and 78/K to the R9.1 RAPD profile, 89/K, 90 K, and 91/K to the R9.3 RAPD profile, as well as 13/K, 14/K, and 16/K to the R10.3 RAPD profile. From the same individual were also received the 23/K and 25/K isolates with different RAPD profiles, the R10.2 and R10.3, respectively. Moreover, two isolates, 32/K and 35/K, isolated from the same goat showed two different RAPD profiles, R9.2 and R5, respectively. All RAPD profiles defined for the tested caprine *T. pyogenes* isolates were presented in Fig. 1 and Table 1.

The dendrogram analysis carried out at the 85% similarity level showed eight clusters (designated from A to H) and two single isolates with RAPD profiles not grouped in any cluster (Fig. 1). The most numerous clusters, H and G, grouped 18 and 12 isolates, respectively. In the cluster H, three different RAPD profiles were observed with 85.6% of similarity. In cluster G, also three various RAPD profiles with 88.1% of similarity were noted. Cluster B contained four isolates (72/K, 73/K, 74/K, and 75/K) with the RAPD profile R2 which were isolated from one individual. Five clusters (A, C, D, E, and F) grouped three isolates, each with a similarity from 85.7% to 100%.

A relatively high discrimination index (D = 0.929) was obtained for RAPD typing of caprine *T. pyogenes* isolates in this study.

The analysis of *T. pyogenes* isolates obtained from animals kept in herd I (*n* = 21) showed that they represented 12 different RAPD profiles. The isolates belonging to three RAPD profiles, R10.2, R10.3 and R9.2, remained in herd I for two to four years of the study period. The isolates with the RAPD profile R10.2 noted in herd I in 2015 (isolates 23/K and 24/K), were also obtained in 2016 (isolate 60/K), 2018 (isolate 80/K) and 2019 (isolate 79/K). The isolates with the RAPD profile R10.3 were observed in this herd in 2015 (isolate 25/K) and 2016 (isolates 12/K, 13/K, 14/K, 15/K, 16/K, 17/K, and 33/K), as well as the isolates with the RAPD profile R9.2 were isolated in 2016 (isolate 32/K) and 2018 (isolate 67/K). In 2016, the highest number of goats from herd I were tested, and *T. pyogenes* with the RAPD profile R10.3 (5/12; 41.7%; CI 95%: 15.2–72.3%) were predominant. Interestingly, in the case of two goats, the *T. pyogenes* isolates with two different RAPD profiles were isolated in 2015 (isolates 23/K and 25/K) and 2016 (isolates 32/K and 35/K). The distribution of individual RAPD profiles over the years of the study in herd I was shown in Additional file 2. In herd II, two isolates of different RAPD profiles were obtained from two tested goats. The isolates of three variable RAPD profiles were also observed in herd III in two goats in 2017 and one individual in 2018.

## Discussion

In many parts of the world, including Asia, Africa, and South America, goat farming is an important branch of the livestock industry. The production of milk and various dairy products plays the main role in the economic development of the goat sector, while meat production is considerably less important [15]. Also, in Poland, goat farming has become increasingly popular [16]. Due to the growing economic value of goats, disease control and maintaining health are relevant.

The occurrence of infections caused by *T. pyogenes* in small ruminants is still poorly known. The results of our study reiterate that *T. pyogenes* is an important pathogen involved in infections in goats. Additionally, it should be mentioned that in this study, this pathogen was isolated as the main etiological agent of the infection. In the available literature, some infections caused by this pathogen in goats were reported. The caprine *T. pyogenes* isolates were obtained from goats with pathologies of respiratory and reproductive tracts, lymph nodes and ocular infections, as well as arthritis and encephalitis [2, 5, 14, 17]. Nevertheless, udder lesions, such as mastitis caused by *T. pyogenes*, are one of the most frequent infectious diseases in goats [2]. Polveiro et al. (2020) noted that *T. pyogenes* occurred in many milk samples taken from goats with persistent mastitis [18]. Moreover, this pathogen was isolated from the lungs of a goat with septicemia [3]. In goat kids, *T. pyogenes* was isolated from abscesses of lungs, liver and heart [6]. The clinical examination confirmed that this bacterium was the major etiological agent of a severe cardiopulmonary disease, which led to the death of goats [6]. Similarly, in our study, we also obtained the vast majority of isolates from the respiratory (48/104; 46.2%) and reproductive tracts (19/104; 18.3%) infections. It should be noted that especially a large number of them were isolated from the lungs, bronchus and udder. Thus, the current investigation reiterates the role of this bacterium in disorders of the respiratory and reproductive tracts in goats. Moreover, the isolation of *T. pyogenes* from the abscesses of various lymph nodes showed that this pathogen may be a potential etiological agent causing infections similar to that related to *C. pseudotuberculosis* [19] and *Staphylococcus aureus* subsp. *anaerobius* [20]; however, in the case of those diseases in goats, mainly superficial nodes are affected.

The knowledge about the occurrence of virulence factors is one of the most relevant aspects of pathogen characterization. The main virulence factor of *T. pyogenes* is PLO, the cytotoxin responsible for lysis of host cells. Until now, gene encoding PLO has been noted in all *T. pyogenes* isolates of different origins, including goats [3–5, 14]. The colonization of host tissues in the first stage of infection is probably possible due to two neuraminidases, NanH and NanP. The presence of genes encoding both these neuraminidases was observed among all caprine isolates in this study. This result is in accordance with previous research [4]. However, caprine *T. pyogenes* isolates did not always carry both of these genes [5, 14]. The different fimbriae, such as FimA, FimC, FimE and FimG, may be involved in the adhesion to host cells [21]. Genes encoding particular fimbriae subunits occurred with variable frequency among the tested isolates. Our results showed that the *fimA* and *fimC* genes were the most prevalent and the *fimE* gene was observed less frequently. It is consistent with the observation noted by Rogovskyy et al. (2018) that the *fimE* gene is less prevalent among isolates obtained from small ruminants [14]. Thus, it appears that FimA and FimC are involved mainly in the adhesion step during *T. pyogenes* infections in goats. In addition, neither of the tested isolates carried the *fimG* gene, while it was detected rarely in other studies [4, 14]. The CbpA is essential in the adhesion to collagen-rich tissues [22]. None of the tested caprine *T. pyogenes* isolates carried the gene encoding this virulence factor. The absence of this gene among isolates from small ruminants was previously reported [3–5, 14]. Among the tested isolates, we observed different virulence genotypes with five or more virulence genes. Interestingly, the genotype containing the *plo*, *nanH*, *nanP*, *fimA,* and *fimC* genes, observed as dominant among tested isolates, was reported by Rogovskyy et al. (2018) as a particular for isolates from small ruminants [14]. Interestingly, both isolates collected from the nasal cavity of animals without any pathological changes also carried a considerable number of virulence genes. Thus, the presence of virulence genes may also be observed in cases of *T. pyogenes* isolates from healthy animals. However, this observation should be confirmed by the study on a larger number of isolates obtained from clinically healthy goats.

It was revealed that bacteria in biofilms, including *T. pyogenes*, were more resistant to antimicrobials than planktonic cells [23]. Due to this, biofilm formation may be one of the important mechanisms determining the resistance of pathogenic bacteria and contributing to antimicrobial therapy failures. Furthermore, biofilm-forming *T. pyogenes* isolates were outlined as relevant contributors to mastitis in dairy cattle [7]. In our study, all tested isolates were able to form biofilm in in vitro conditions, however, with variable intensity. To the best of our knowledge, this study provides the first data about biofilm formation among caprine *T. pyogenes* isolates. Thus far, the ability to biofilm formation has been observed among *T. pyogenes* isolates from cattle and forest musk deer [7–9]. Ozturk et al. (2016) reported that biofilm formation was found in 88.6% of *T. pyogenes* bovine isolates, including all isolates recovered from milk from cattle with mastitis in Turkey [8]. Similarly, in China, 90% of *T. pyogenes* isolates from milk from cattle with mastitis were able to form biofilm [7]. In addition, the majority of *T. pyogenes* isolated from abscesses from forest musk deer (94.4%) were also positive for biofilm formation [9]. Our results classified almost three fourth of tested caprine *T. pyogenes* isolates as strong biofilm formers, whereas moderate and weak biofilm formers were represented by roughly 20% and 8% of isolates, respectively. So far, in the case of *T. pyogenes*, the intensity of biofilm forming has been evaluated only in one study, conducted on bovine isolates [7]. In the work mentioned above, a significant number of tested isolates were recognized as low-grade biofilm formers (54%), whereby fewer of them were found to be highly biofilm positive (36%) [7]. Additionally, it should be mentioned that in contrast to our results, the single isolates unable to form biofilm were noted in other studies [7–9].

The *luxS* gene associated with the regulation of biofilm formation was found in all tested *T. pyogenes* isolates from goats in this study. The presence of this gene was also observed in the genome sequence of the caprine *T. pyogenes* strain available in the GenBank database under accession number CP012649 [24]. As the intensity of biofilm formation varied among the tested *T. pyogenes* isolates and the *luxS* gene was detected in all of them, other determinants may be associated with this property in the pathogen. Zhang et al. (2021) noted that the expression of two TatD DNases (TatD DNase 825 and TatD DNase 960) may be involved in the virulence of *T. pyogenes*, including biofilm formation [25].

In our previous studies, the optimized RAPD-PCR method with the M13 primer was efficiently used for typing *T. pyogenes* isolates obtained from various animal species, including several isolates of caprine origin [12, 13]. The effectiveness of this method to assess the genetic diversity of *T. pyogenes* isolates was confirmed in this research. Consequently, the high D value, relevant for the reliable interpretation of discrimination levels of the typing method, was obtained. The present study indicates that *T. pyogenes* isolates causing infections in goats in Poland are significantly diverse. However, in our previous study, we observed higher genetic diversity of *T. pyogenes* isolates obtained from wild ruminants [12]. In this research, a relatively high number of different RAPD profiles was observed among tested caprine isolates. The same RAPD profile was observed among isolates from the same individuals but from different clinical materials. Interestingly, in two cases, other RAPD profiles were found in *T. pyogenes* isolates from various lesions in the same goats. Due to this fact, it seems that sometimes different disorders may be associated with other *T. pyogenes* strains in one individual. However, in many cases, the same RAPD profile was observed among isolates from various individuals. Moreover, the majority of isolates from a particular herd were highly related and grouped in the same cluster. Thus, the possible transmission of a *T. pyogenes* strain among goats kept in the same herd should be considered. It is necessary to mention that in addition to the earlier investigation by Rogovskyy et al. [14], where 19 caprine isolates of *T. pyogenes* were molecularly and phenotypically characterized, the current study provides an additional set of data (51 isolates) regarding the genetic diversity of caprine isolates.

This study has some limitations. One of them is associated with the use of the RAPD-PCR method. It is considered that some other fingerprinting methods, such as the pulsed field gel electrophoresis (PFGE), are more suitable and detailed for the genetic differentiation of bacterial strains. However, PFGE is lengthy, laborious, and requires special equipment, which may sometimes make it difficult to use [26]. On the other hand, the whole genome sequencing (WGS) of isolates would certainly provide valuable data on differentiations among strains, including more information about antimicrobial resistance and virulence factors [26]. While it should be mentioned that WGS is still quite an expensive method, especially when testing a large number of strains. Therefore, the use of RAPD-PCR as the first step in understanding the relationship among bacterial strains seems to be justified. According to the results obtained from this study, the genetic diversity of isolates from goats is lower than that observed among free-living animals [12]. It should be mentioned that the number of herds from which *T. pyogenes* isolates were obtained was fairly limited in our research. Moreover, information about age, sex and breed, which could be important from the epidemiological point of view, was lacking. Examination of a higher number of strains isolated from different herds might provide additional data regarding infections caused by this pathogen in small ruminants. Therefore, it seems that the possibility of the spread of *T. pyogenes* strains among individuals from the same herd, as well as between herds, should be observed.

## Conclusion

The results of this study contribute to understanding the epidemiology of the *T. pyogenes* infections in goats. This bacterium should be considered one of the important etiological agents of infections in goats, especially disorders of respiratory and reproductive tracts, as well as a cause of abscesses. It is known that the pathogenicity of caprine *T. pyogenes* strains seems to be associated with different virulence factors. However, both neuraminidases, as well as FimA and FimC, may appear to be more significant determinants of virulence. The genetic diversity analysis showed that the isolates from the same individual usually had the same and less often variable RAPD profiles. Moreover, the presence of isolates characterized by the same RAPD profile in different goats in this study suggests a possible *T. pyogenes* transmission between animals within a herd or between herds.

## Methods

### Material collection

Between April 2015 and May 2023, various clinical specimens (*n* = 1146) collected from goats with purulent lesions were subjected to a bacteriological examination at the Microbiological Diagnostic Laboratory, Department of Preclinical Sciences, Institute of Veterinary Medicine, Warsaw University of Life Sciences-SGGW in Poland. The following numbers of samples were tested each year: 2015 (*n* = 147), 2016 (*n* = 210), 2017 (*n* = 287), 2018 (*n* = 163), 2019 (*n* = 47), 2020 (*n* = 73), 2021 (*n* = 57), 2022 (*n* = 127), and 2023 (*n* = 35). Tested specimens were collected from animals from eight goat herds in Poland (Table 3). In the case of 488 studied specimens, detailed information about their origin was unavailable, although they were known to have come from goat herds from all over Poland.

**Table 3:** The number of clinical specimens and herds from which clinical specimens were collected

Herd number	Number of	
	clinical specimens collected from diseased goats (*n* = 1146)	specimens collected from clinically healthy goats (*n* = 306)
I	467	16
II	80	-
III	82	106
IV	6	-
V	1	-
VI	1	-
VII	9	-
VIII	12	-
IX	-	26
X	-	9
XI	-	20
XII	-	17
XIII	-	33
unknown	488	79

In addition, in 2018, 306 swabs obtained from clinically healthy goats from seven herds (Table 3) were examined for *T. pyogenes* presence to assess the pathogen carriage. In the case of 79 studied specimens, data about herds were unavailable. All herds were located in Poland. These swabs were collected from the nasal cavity (*n* = 141), groin (*n* = 120), rectum (*n* = 30), and oral cavity (*n* = 15).

### Isolation and identification of *T. pyogenes*

All specimens were cultured on Columbia Agar supplemented with 5% sheep blood (CAB) (Graso Biotech, Starogard Gdański, Poland) at 37 °C in 5% CO_2_ atmosphere for 48 h. *T. pyogenes* isolates were identified based on the phenotypic properties, including the colony morphology (especially the presence of the β-hemolysis zone on CAB), the cell morphology in the Gram-stained smears, the effect of the CAMP test with the *S. aureus* ATCC®25,923 reference strain and the negative catalase activity test. The initial identification of *T. pyogenes* isolates was confirmed by the detection of the *plo* gene, recognized as specific of this species, by PCR [12]. The isolates were stored at -20 °C in the Tryptic Soy Broth (TSB) (Graso Biotech, Starogard Gdański, Poland) with 20% glycerol (*v/v*) for further testing.

### Biofilm formation assay

The biofilm formation in vitro was performed by the microtiter assay as previously described by Stepanović et al. (2007) with some modifications [27]. Briefly, each *T. pyogenes* strain was cultured on CAB for 48 h at 37 °C in 5% CO_2_ atmosphere. After incubation, several bacterial colonies were transferred to an ampule containing 2 mL of NaCl 0.85% Medium (bioMérieux, France) to match the turbidity to the 2 McFarland standard. Then, the bacterial suspension was diluted in TSB to the final concentration of bacterial inoculum, approximately 10^6^ CFU/mL, and 200 μL was added into three wells of a sterile 96-well flat-bottom microtiter plate (Sarstedt, Nümbrecht, Germany). The sterile TSB was used as a negative control. The plate was incubated at 37 °C for 48 h in 5% CO_2_ atmosphere without agitation.

The crystal violet (CV) staining method was used to quantify biofilm biomass. After incubation, the culture medium was carefully removed from each well and the plate was washed three times with 200 μL of phosphate-buffered saline (PBS) to remove unattached bacterial cells. The plate was allowed to dry at approximately 60 °C for 15 min to fix the biofilm. Then, the adherent cells were stained with 150 μL of 0.5% (*v/v*) CV for 15 min at room temperature. The excess stain was removed, and the plate was washed three times with 200 μL of tap water and dried for 15 min at room temperature. Subsequently, 150 μL of 96% ethanol was added to each well for 30 min to dissolve the CV at room temperature. The well contents were aspirated and transferred to a new plate. The microplate reader Epoch (BioTek Instruments, Winooski, VT, USA) was used to determine the sample absorbance (optical density) at the wavelength of 570 nm (OD_570_). Then, the arithmetic mean and standard deviation (SD) of OD_570_ were calculated for each tested caprine *T. pyogenes* isolate. All experiments had three technical replicates, as well as were performed in triplicate, thus finally, for each strain, nine replications were reported.

The mean OD_570_ values of the negative control (sterile TSB) were used as a standard for the classification of the biofilm-forming caprine *T. pyogenes* isolates, according to Stepanović et al. (2007) [27]. The cut-off OD (OD_c_) was calculated as three SD above the mean OD of the negative control. Each of the isolates were classified into the following categories: non-biofilm former (OD_570_ ≤ OD_c_), weak biofilm former (OD_c_ < OD_570_ ≤ 2 × OD_c_), moderate biofilm former (2 × OD_c_ < OD_570_ ≤ 4 × OD_c_) or strong biofilm former (4 × OD_c_ < OD_570_).

### Genomic DNA isolation

To extract genomic DNA, each *T. pyogenes* isolate was cultured for 48 h at 37 °C in 5% CO_2_ atmosphere on CAB. After the incubation, several colonies were picked, suspended in 500 μL of distilled water and the suspension was centrifuged at 12,000 × *g* for 8 min. The pellet was used for DNA extraction with the Genomic Mini AX Bacteria + Spin kit (A&A Biotechnology, Gdańsk, Poland) according to the manufacturer's protocol. The elution was performed using 100 μL of the elution buffer E heated to 65 °C. The concentration and purity of the extracted genomic DNA were determined with the spectrophotometer NanoDrop® ND-1000 (Thermo Fisher Scientific, Waltham, MA, USA). The genomic DNA samples were stored at -20 °C until testing.

### Detection of virulence genes

The presence of eight genes encoding putative virulence factors (*plo*, *nanH*, *nanP*, *cbpA*, *fimA*, *fimC*, *fimE*, *fimG*) and one gene regulating the biofilm formation (*luxS*) of *T. pyogenes* was investigated by PCR. Each reaction mixture (25 µL final volume) contained: 8.5 µL of nuclease-free water (Thermo Fisher Scientific, Waltham, MA, USA), 12.5 µL of DreamTaq Green PCR Master Mix (2X) (Thermo Fisher Scientific, Waltham, MA, USA), 10 pmol of each primer (Genomed, Warsaw, Poland) and 70–80 ng of the genomic DNA. The thermal cycling conditions included initial denaturation at 95 °C for 3 min followed by 35 cycles of DNA denaturation at 95 °C for 1 min, annealing for 1 min at variable temperatures listed in Table 4, and extension at 72 °C for 3 min with a final extension step of 72 °C for 7 min.

**Table 4:** Primer sequences and PCR conditions used to detect virulence factors and the gene regulating the biofilm formation in the caprine *T. pyogenes* isolates

Virulence factor	Target gene	Primer sequence (5’-3’)	Annealing temperature (°C)	Amplicon size (bp)	Reference
Pyolysin	*plo*	F–TCATCAACAATCCCACGAAGAG R–TTGCCTCCAGTTGACGCTTT	60	150	[28]
Neuraminidase H	*nanH*	F–CGCTAGTGCTGTAGCGTTGTTAAGT R–CCGAGGAGTTTTGACTGACTTTGT	60	781	[28]
Neuraminidase P	*nanP*	F–TTGAGCGTACGCAGCTCTTC R–CCACGAAATCGGCCTTATTG	60	150	[28]
Collagen-binding protein	*cbpA*	F–GCAGGGTTGGTGAAAGAGTTTACT R–GCTTGATATAACCTTCAGAATTTGCA	60	124	[28]
Fimbriae A	*fimA*	F–CACTACGCTCACCATTCACAAG R–GCTGTAATCCGCTTTGTCTGTG	57	605	[28]
Fimbriae C	*fimC*	F–TGTCGAAGGTGACGTTCTTCG R–CAAGGTCACCGAGACTGCTGG	60	843	[28]
Fimbriae E	*fimE*	F–GCCCAGGACCGAGAGCGAGGGC R–GCCTTCACAAATAACAGCAACC	55	775	[28]
Fimbriae G	*fimG*	F–ACGCTTCAGAAGGTCACCAGG R–ATCTTGATCTGCCCCCATGCG	57	929	[28]
S-ribosylhomocysteine lyase	*luxS*	F–CGAGTCCTTTAACCTCGACC R–TCATGAGTACACCTCTCCCC	53	445	This study

The primer set to detect the *luxS* gene was designed using the PCR Primer Design (https://eurofinsgenomics.eu/en/ecom/tools/pcr-primer-design/, accessed on 21 February 2023) and checked using the Oligo Analysis Tool (https://www.eurofinsgenomics.eu/en/ecom/tools/oligo-analysis/, accessed on 21 February 2023). The sequence of *luxS* obtained from the complete genome sequence of the *T. pyogenes* strain isolated from a goat (GenBank accession no. CP012649) was used for the primer development.

Amplification products were recognized by electrophoresis (90 V for 45 min) in 1% (*w/v*) agarose gel in Tris–Acetate-EDTA (TAE) buffer with Midori Green DNA Stain (Nippon Genetics, Düren, Germany), visualized and analysed using a Gel Doc™ EZ Imaging System with Image Lab Software (version 5.2.1) (Bio-Rad, Hercules, USA). The GeneRuler 100 bp Plus DNA Ladder (Thermo Fisher Scientific, Waltham, MA, USA) was used for estimating the molecular size of obtained bands.

### RAPD-PCR typing of caprine *T. pyogenes* isolates

The RAPD-PCR typing of caprine *T. pyogenes* isolates was performed with the M13 primer (5’-GAGGGTGGCGGTTCT-3’) (Eurofins Genomics, Ebersberg, Germany) according to our previous studies [12, 13]. Each reaction mixture contained: 12.5 µL of DreamTaq Green PCR Master Mix (2X) (Thermo Fisher Scientific, Waltham, MA, USA), 3.5 mM MgCl_2_ (Thermo Fisher Scientific, Waltham, MA, USA), 0.8 mM of each dNTP (Thermo Fisher Scientific, Waltham, MA, USA), 20 pmol of the M13 primer, 20 ng of the genomic DNA, and nuclease-free water (Thermo Fisher Scientific, Waltham, MA, USA) up to a final volume of 25 µL. The thermal cycling conditions were as follows: initial denaturation at 94 °C for 3 min followed by 40 cycles of DNA denaturation at 94 °C for 30 s, annealing for 30 s at 45 °C and extension at 72 °C for 1 min with a final extension step of 72 °C for 7 min. Amplification products were recognized by electrophoresis (90 mA for 3.5 h) in 2% (*w/v*) agarose gel in Tris–Borate-EDTA (TBE) buffer with Midori Green DNA Stain (Nippon Genetics, Düren, Germany), visualized and analyzed using a Gel Doc™ EZ Imaging System with Image Lab Software (version 5.2.1) (Bio-Rad, Hercules, USA). The GeneRuler 1 kb DNA Ladder (Thermo Fisher Scientific, Waltham, MA, USA) was used for estimating the molecular size of obtained bands.

The analysis was performed using the BioNumerics software version 7.6 (Applied Maths, Sint-Martens-Latem, Belgium). The Unweighted Pair Group Method with Arithmetic Mean (UPGMA) using Dice similarity coefficient with optimization and position tolerance set at 1% was used in the cluster analysis. Isolates were clustered using an 85% homology cut-off, above which isolates were considered closely related and assigned to the same cluster.

The discrimination index (D) was calculated to evaluate the discriminatory power of the used method of typing *T. pyogenes* isolates obtained from goats [29].

### Statistical analysis

Categorical data were expressed as counts (*n*) and percentages (%). The 95% confidence intervals (CI 95%) for proportions were calculated using the Wilson score method [30]. Percentages were compared between groups using the maximum likelihood G test. OD values were presented as the arithmetic mean and standard deviation (SD) when appropriate.

## Supplementary Information

Additional file 1. The biofilm formation properties of caprine *Trueperella pyogenes *isolates (*n*=51). For each isolate, the values of nine replications are reported.

Additional file 2. The occurrence of RAPD profiles of caprine *T. pyogenes* isolates in herd I in the period from 2015 to 2019.

## Data Availability

The datasets supporting the conclusions of this article are included within the article and its additional file.

## Abbreviations

ATCC: American Type Culture Collection
CAB: Columbia Agar supplemented with 5% sheep blood
CAMP: Christie–Atkins–Munch-Peterson
CbpA: Collagen-binding protein
CI: Confidence intervals
CV: Crystal violet
D: Discrimination index
FimA: Fimbriae subunit A
FimC: Fimbriae subunit C
FimE: Fimbriae subunit E
FimG: Fimbriae subunit G
NanH: Neuraminidase H
NanP: Neuraminidase P
OD: Optical density
PBS: Phosphate-buffered saline
PFGE: Pulsed field gel electrophoresis
PLO: Pyolysin
RAPD-PCR: Random Amplified Polymorphic DNA-Polymerase Chain Reaction
SD: Standard deviation
TAE: Tris–Acetate-EDTA
TBE: Tris–Borate-EDTA
TSB: Tryptic Soy Broth
UPGMA: Unweighted Pair Group Method with Arithmetic Mean
