# Supplementary material for: Genetic diversity and virulence properties of caprine Trueperella pyogenes isolates
Source: BMC Vet Res. 2024 Sep 6;20:395. doi: 10.1186/s12917-024-04262-x (PMC11378509; doi:10.1186/s12917-024-04262-x)
Supplement: Supplementary file 1 — Additional file 1. The biofilm formation properties of caprine Trueperella pyogenes isolates (n=51). For each isolate, the values of nine replications are reported. [file 12917_2024_4262_MOESM1_ESM.docx]

**Additional file 1.** The biofilm formation properties of caprine *Trueperella pyogenes* isolates (*n*=51). For each isolate the values of nine replications are reported.

| Isolate designation | Absorbance (optical density) at the wavelength of 570 nm | | | | | | | | | Arithmetic mean | Standard deviation | Biofilm formation^*^ |
| --- | --- | --- | --- | --- | --- | --- | --- | --- | --- | --- | --- | --- |
| 12/K | 0.581 | 0.372 | 0.553 | 0.440 | 0.350 | 0.536 | 0.400 | 0.426 | 0.496 | 0.462 | 0.083 | SBF |
| 13/K^1^ | 1.229 | 1.227 | 1.050 | 1.304 | 1.025 | 1.059 | 0.980 | 1.393 | 1.089 | 1.151 | 0.142 | SBF |
| 14/K^1^ | 3.457 | 3.369 | 3.364 | 3.454 | 3.446 | 3.403 | 3.462 | 3.470 | 3.449 | 3.430 | 0.041 | SBF |
| 15/K | 0.088 | 0.100 | 0.085 | 0.101 | 0.087 | 0.096 | 0.133 | 0.149 | 0.092 | 0.103 | 0.022 | WBF |
| 16/K^1^ | 0.457 | 0.496 | 0.813 | 0.681 | 0.697 | 0.769 | 0.781 | 0.832 | 0.568 | 0.677 | 0.139 | SBF |
| 17/K | 0.585 | 0.398 | 0.345 | 0.333 | 0.564 | 0.476 | 0.479 | 0.446 | 0.249 | 0.431 | 0.110 | SBF |
| 18/K^2^ | 0.980 | 1.063 | 1.046 | 1.323 | 1.238 | 1.322 | 1.196 | 1.010 | 1.377 | 1.173 | 0.151 | SBF |
| 23/K^3^ | 0.582 | 0.910 | 0.944 | 0.800 | 0.886 | 0.983 | 0.644 | 0.560 | 0.700 | 0.779 | 0.162 | SBF |
| 24/K | 1.093 | 1.086 | 0.969 | 0.755 | 0.599 | 0.791 | 0.799 | 0.913 | 1.002 | 0.890 | 0.166 | SBF |
| 25/K^3^ | 0.593 | 0.806 | 0.929 | 0.857 | 0.873 | 0.802 | 1.037 | 0.836 | 0.649 | 0.820 | 0.134 | SBF |
| 29/K | 0.207 | 0.447 | 0.238 | 0.172 | 0.775 | 0.489 | 0.323 | 0.574 | 0.585 | 0.423 | 0.204 | SBF |
| 32/K^4^ | 0.113 | 0.084 | 0.469 | 0.110 | 0.095 | 0.337 | 0.546 | 0.609 | 0.399 | 0.307 | 0.211 | MBF |
| 33/K | 0.183 | 0.255 | 0.432 | 0.285 | 0.268 | 0.299 | 0.455 | 0.420 | 0.413 | 0.334 | 0.097 | MBF |
| 35/K^4^ | 0.085 | 0.069 | 0.068 | 0.074 | 0.068 | 0.164 | 0.195 | 0.146 | 0.281 | 0.128 | 0.075 | WBF |
| 50/K | 0.245 | 0.197 | 0.296 | 0.316 | 0.302 | 0.239 | 0.144 | 0.480 | 0.251 | 0.274 | 0.096 | MBF |
| 55/K^2^ | 0.537 | 0.454 | 0.546 | 0.729 | 0.545 | 0.718 | 0.647 | 0.548 | 0.964 | 0.632 | 0.154 | SBF |
| 57/K | 0.712 | 0.631 | 0.644 | 0.731 | 0.687 | 0.671 | 0.497 | 0.537 | 0.507 | 0.624 | 0.089 | SBF |
| 58/K | 0.769 | 0.898 | 0.755 | 0.790 | 1.028 | 0.884 | 1.148 | 0.994 | 1.146 | 0.935 | 0.153 | SBF |
| 59/K | 0.383 | 0.324 | 0.243 | 0.298 | 0.324 | 0.471 | 0.586 | 0.515 | 0.357 | 0.389 | 0.112 | SBF |
| 60/K | 0.493 | 0.429 | 0.508 | 0.521 | 0.495 | 0.430 | 0.506 | 0.417 | 0.352 | 0.461 | 0.057 | SBF |
| 61/K | 0.505 | 0.478 | 0.641 | 0.754 | 0.668 | 0.655 | 0.504 | 0.628 | 0.479 | 0.590 | 0.100 | SBF |
| 62/K | 0.714 | 0.460 | 0.615 | 0.789 | 0.650 | 0.670 | 0.557 | 0.587 | 0.464 | 0.612 | 0.109 | SBF |
| 63/K | 0.583 | 0.870 | 0.890 | 1.127 | 0.844 | 0.738 | 0.764 | 1.161 | 0.624 | 0.845 | 0.199 | SBF |
| 64/K | 0.169 | 0.095 | 0.186 | 0.145 | 0.166 | 0.100 | 0.109 | 0.134 | 0.090 | 0.133 | 0.036 | WBF |
| 65/K | 0.192 | 0.192 | 0.132 | 0.152 | 0.150 | 0.154 | 0.128 | 0.157 | 0.193 | 0.161 | 0.025 | WBF |
| 66/K | 1.270 | 1.098 | 1.190 | 1.060 | 1.259 | 1.196 | 1.197 | 1.145 | 1.192 | 1.179 | 0.068 | SBF |
| 67/K | 0.815 | 0.961 | 1.072 | 0.853 | 0.704 | 1.051 | 0.904 | 0.841 | 0.675 | 0.875 | 0.138 | SBF |
| 68/K | 0.302 | 0.324 | 0.348 | 0.296 | 0.348 | 0.347 | 0.484 | 0.386 | 0.363 | 0.355 | 0.056 | MBF |
| 69/K | 2.637 | 2.371 | 2.452 | 2.489 | 2.640 | 2.675 | 2.729 | 2.635 | 2.523 | 2.572 | 0.118 | SBF |
| 72/K^5^ | 0.261 | 0.333 | 0.241 | 0.319 | 0.350 | 0.333 | 0.376 | 0.351 | 0.588 | 0.350 | 0.099 | MBF |
| 73/K^5^ | 0.406 | 0.478 | 0.660 | 0.311 | 0.456 | 0.519 | 0.443 | 0.437 | 0.678 | 0.488 | 0.117 | SBF |
| 74/K^5^ | 0.497 | 0.493 | 0.391 | 0.380 | 0.546 | 0.676 | 0.544 | 0.680 | 0.415 | 0.514 | 0.111 | SBF |
| 75/K^5^ | 0.520 | 0.467 | 0.471 | 0.635 | 0.554 | 0.456 | 0.550 | 0.597 | 0.654 | 0.545 | 0.070 | SBF |
| 76/K^6^ | 0.265 | 0.272 | 0.300 | 0.283 | 0.305 | 0.294 | 0.294 | 0.293 | 0.301 | 0.289 | 0.014 | MBF |
| 77/K^6^ | 0.264 | 0.255 | 0.293 | 0.310 | 0.223 | 0.261 | 0.273 | 0.230 | 0.222 | 0.259 | 0.031 | MBF |
| 78/K^6^ | 0.300 | 0.228 | 0.201 | 0.185 | 0.281 | 0.253 | 0.244 | 0.198 | 0.256 | 0.238 | 0.039 | MBF |
| 79/K | 0.351 | 0.555 | 0.436 | 0.463 | 0.154 | 0.143 | 0.529 | 0.192 | 0.339 | 0.351 | 0.158 | MBF |
| 80/K | 0.380 | 0.415 | 0.469 | 0.460 | 0.401 | 0.440 | 0.487 | 0.409 | 0.452 | 0.435 | 0.035 | SBF |
| 81/K | 0.768 | 0.788 | 1.256 | 1.010 | 1.236 | 0.860 | 0.972 | 1.057 | 1.197 | 1.016 | 0.187 | SBF |
| 82/K | 1.381 | 1.310 | 1.256 | 1.421 | 1.359 | 1.199 | 1.533 | 1.560 | 1.290 | 1.368 | 0.121 | SBF |
| 83/K | 1.107 | 1.167 | 1.001 | 1.237 | 1.044 | 0.756 | 0.757 | 0.913 | 0.843 | 0.981 | 0.175 | SBF |
| 84/K | 1.341 | 1.257 | 1.311 | 1.209 | 0.967 | 0.972 | 0.983 | 1.190 | 1.078 | 1.145 | 0.149 | SBF |
| 85/K | 0.772 | 0.653 | 0.684 | 0.735 | 0.635 | 0.585 | 0.705 | 0.576 | 0.732 | 0.675 | 0.068 | SBF |
| 86/K | 1.166 | 0.699 | 0.666 | 1.447 | 0.960 | 1.653 | 1.597 | 1.651 | 1.629 | 1.274 | 0.412 | SBF |
| 87/K | 1.287 | 1.275 | 1.260 | 1.471 | 1.355 | 1.364 | 1.371 | 1.243 | 1.258 | 1.320 | 0.075 | SBF |
| 88/K | 0.608 | 0.729 | 0.511 | 0.525 | 0.470 | 0.503 | 0.667 | 0.782 | 0.795 | 0.621 | 0.127 | SBF |
| 89/K^7^ | 1.849 | 2.298 | 2.419 | 2.220 | 2.449 | 1.951 | 2.001 | 1.845 | 1.906 | 2.104 | 0.244 | SBF |
| 90/K^7^ | 3.173 | 2.574 | 2.443 | 3.129 | 2.243 | 2.659 | 2.712 | 2.773 | 3.083 | 2.754 | 0.321 | SBF |
| 91/K^7^ | 2.609 | 2.625 | 2.782 | 3.485 | 2.640 | 3.378 | 3.301 | 3.020 | 2.409 | 2.917 | 0.392 | SBF |
| 92/K | 1.319 | 0.959 | 0.894 | 1.117 | 0.950 | 0.992 | 1.154 | 1.280 | 1.091 | 1.084 | 0.149 | SBF |
| 93/K | 0.334 | 0.469 | 0.177 | 0.281 | 0.251 | 0.411 | 0.232 | 0.323 | 0.507 | 0.332 | 0.111 | MBF |

^1-7^*T. pyogenes* isolates from the same goats but from different clinical samples.

^*^Biofilm formation categorization: strong biofilm former (SBF), moderate biofilm former (MBF), weak biofilm former (WBF).
