# Supplementary figures and images for: Genetic diversity and virulence properties of caprine Trueperella pyogenes isolates
Source: BMC Vet Res. 2024 Sep 6;20:395. doi: 10.1186/s12917-024-04262-x (PMC11378509; doi:10.1186/s12917-024-04262-x)

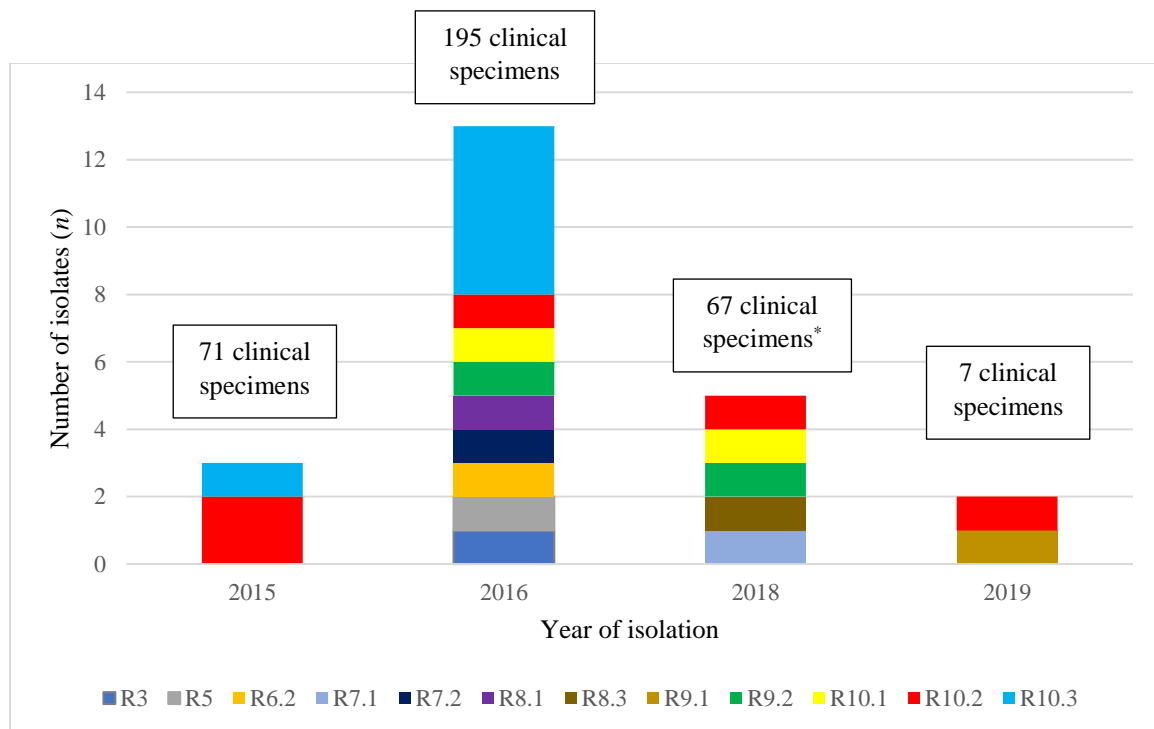

\*Including 16 clinical specimens obtained from clinically healthy goats.

Supplement: Supplementary file 2 — Additional file 2. The occurrence of RAPD profiles of caprine T. pyogenes isolates in herd I in the period from 2015 to 2019. [file 12917_2024_4262_MOESM2_ESM.pdf]
